# Editorial: Concepts and Experiences in Framing, Integration and Evaluation of One Health and EcoHealth

**DOI:** 10.3389/fvets.2019.00155

**Published:** 2019-05-24

**Authors:** Simon R. Rüegg, Sandra C. Buttigieg, Flavie L. Goutard, Aurélie Binot, Serge Morand, Séverine Thys, Hans Keune

**Affiliations:** ^1^Section of Epidemiology, Vetsuisse Faculty, University of Zurich, Zurich, Switzerland; ^2^Health Services Management, Faculty of Health Sciences, Msida, Malta; ^3^Centre de Coopération Internationale en Recherche Agronomique pour le Développement (CIRAD), Montpellier, France; ^4^Centre National de la Recherche Scientifique (CNRS), Paris, France; ^5^Department of Virology, Parasitology, and Immunology, Faculty of Veterinary Medicine, Ghent University, Ghent, Belgium; ^6^Belgian Biodiversity Platform - Research Institute Nature & Forest (INBO), Brussels, Belgium; ^7^Department of Primary and Interdisciplinary Care Antwerp, Faculty of Medicine and Health Sciences, University of Antwerp, Wilrijk, Belgium

**Keywords:** one health (OH), evaluation, integrated approaches to health, transdisciplinarity, public health, veterinary public health, ecosystem health, health policy

The work presented in this research topic shows that the context of initiatives in One Health (and other integrated approaches to health) is crucial and plays an important role for their implementation. Consequently, conceptualization, as well as evaluation should take into account the wider system in which such endeavors are set and cater for multiple perspectives. The featured manuscripts provide a wealth of concepts for implementation and evaluation of integrated approaches to health, which are exemplified with real cases.

Collaborative approaches across disciplines and sectors are recognized as necessary to address *wicked* problems, which prove difficult to solve singlehandedly. The recent financial, economic, social, environmental and health crises further added to the challenges of providing tangible solutions to these problems. In the health domains, classical examples are antibiotic resistance or outbreaks of highly infectious diseases, e.g., Highly Pathogenic Avian Influenza (HPAI), Ebola, Severe Acute Respiratory Syndrome (SARS), Zika virus disease, which gave fresh impetus to integrated approaches to health. However, it must be recognized that these concepts have a long history and have been named with a variety of terms such as One Health, EcoHealth, Global Health, Planetary Health, Ecological Public Health, Environmental Health, Health in scaled Social-Ecological Systems, or others, depending on the specific perspective. They all share the characteristics of integrating knowledge and skills from multiple stakeholders within and across a variety of disciplines and sectors, aiming to collectively find sustainable health solutions.

Lerner and Berg investigated the similarities and differences of three currently very influential holistic concepts: One Health, EcoHealth, and Planetary Health. They found that One Health has been described as either a narrow collaboration of public health and veterinary medicine or as wide-spread interdisciplinary field with a focus on vertebrate health. EcoHealth appeared to emphasize more on including all living creatures down to microscopic levels, while Planetary Health seemed more concerned with human health at global scale. This article documents that despite all being holistic approaches, they emerge from different core values. Interestingly, a bibliometric study investigating studies on dynamic disease modeling, substantiated similar silos previously, even within the One Health community (1). Duboz et al. inferred that the common holism is grounded in systems thinking and enabled by participatory modeling, which is why they advocate for the systematic incorporation of specialists in systems science and social engagement for all integrated approaches to health. The apparent difference in scope of One Health and EcoHealth has been alluded to converge already previously (2), and in their respective contributions, Destoumieux-Garzón et al. and Wilcox et al. elaborate and analyse theories and practical examples to explain this convergence. The first team highlights the value of ecological, evolutionary, and environmental sciences in understanding factors underlying stress responses and developing novel strategies to achieve manageable equilibria and dynamics in ecosystems to foster health for all. The second team shows how the social-ecological systems and resilience theory contribute to the One Health approach by illustrating two examples in the Greater Mekong subregion and their contribution toward the UN Sustainable Development Goals.

Focussing on perceptions from the public, Padda et al. investigated how One Health was conceptualized by regular visitors of the St. Louis Zoo (USA). They demonstrated that in contrast to infectious diseases, which are often cited as field of application for One Health, the zoo public appeared to be primarily concerned by chronic, non-communicable diseases. The findings reflect the epidemiologic transition experienced in that region and underline that zoos, particularly in the industrialized nations, are expected to play an important role in promoting One Health locally. In a different urban setting, Alarcon et al. studied livestock keeping and food supply chains in Nairobi. They showed that very diverse and predominantly small-scale farming in a densely populated environment is a challenge for managing animal health and protecting humans from food-borne diseases. However, they identified critical agents in the system and provided baseline information to develop effective policies.

The next set of manuscripts moved from understanding and conceptualizing to evaluating the implementation of integrated approaches to health. While in the discourse above, systems theory is an important tenet, Valeix focussed on the concept of “integration” as a pivotal aspect of implementing One Health approaches. She framed integration as “complementary to” or “composed of” collaboration, cooperation and coordination. The framework she developed probes the social dimensions and power dynamics among professional participants that affect One Health implementation. She emphasized the importance of local and national levels for the successful realization of One Health and exemplified the approach for zoonotic disease management in Ghana focussing on the veterinary actors. Rüegg et al. presented the framework developed by the EU COST Action “Network for Evaluation of One Health” (NEOH, https://neoh.onehealthglobal.net), an international, open network with more than 240 participants from 30 countries. It pursued a systems approach to evaluation and comprised a One Health-index and -ratio as a semi-quantitative measure to assess systems thinking, planning, transdisciplinary, and participatory working, sharing and learning infrastructures, as well as adaptive leadership in One Health initiatives. The framework was tested in various case studies from the network: Buttigieg et al., Paternoster et al., Fonseca et al., Hanin et al. and their co-workers evaluated One Health approaches for infectious disease surveillance and control. Comparing the One Health-indices and -ratios revealed that time is a relevant factor for the implementation of such initiatives, with older efforts becoming more holistic. The case study on preventing the misuse of acaricide containers for food and water storage after an animal health intervention underpins this observation, as it shows a very good balance between all six evaluated aspects of One Health and was implemented as a satellite project of a well-established field program. The case study on the Southern African Centre for Infectious Disease Surveillance also indicates that national borders are challenging for the sharing of data. Looking at the evaluations of an academic One Health research program to tackle antimicrobial resistance and a study on obesity in European dogs and their owners suggests that the professional context of a One Health initiative determines much of its capability to implement a holistic approach and that the prevailing competitive mentality in the academic field may pose a serious obstacle to the endeavor. Finally, Radeski et al. demonstrate how the target of improving animal welfare aligns surprisingly well with the One Health concept. All case studies conclude that systems thinking is challenging for many natural scientists but that the NEOH framework is a helpful tool for feedback, accountability and even conception of One Health initiatives.

In the time this special issue was collected, the World Bank published its “operational framework for strengthening human, animal, and environmental public health systems at their interface”(3), the Food and Agriculture Organization (FAO), the World Organization of Animal Health (OIE) and the World Health Organization (WHO) have jointly published “a tripartite guide to addressing zoonotic diseases in countries”(4), and the UN Convention on Biological Diversity (CBD) has issued its “guidance on integrating biodiversity considerations into one health approaches” (5). Also, in other communities the wealth of literature on systemic approaches is growing rapidly and there are countless opportunities to cross-fertilize between different fields of application. This confirms the timeliness of this Research Topic, which we believe, contributes valuable resources for practitioners, policy-makers, and funders. It also highlights a diversity of core values rooted in different systems and temporal–spatial scales that give rise to a gap between policy and practice. Closer examination of the articles raises the concern that nature based benefits as well as cross-cultural perspectives (6) are underrepresented in the multiple One Health narratives and their management. In the search for generic validity, it goes unnoticed that we know very little about the lives of those who experience these complex entanglements between humans, animals, and ecosystems on a daily basis, and whose stewardship is decisive for change to occur. We also suspect that sustained projects foster a more holistic view than short term investigations and that professional idiosyncrasies may hamper integration of knowledge, which both challenge the current academic and research practice. But now that we have paused to consolidate our knowledge and experiences with integrated approaches to health, we shall open our minds to tackle these obstacles.

## Author Contributions

All authors have served as editors of the research topic. SR has written the draft of the editorial and it was amended and revised by the other authors.

### Conflict of Interest Statement

The authors declare that the research was conducted in the absence of any commercial or financial relationships that could be construed as a potential conflict of interest.
